# Excision of a Mammary Gland

**Published:** 1896-08

**Authors:** W. J. Martin

**Affiliations:** Kankakee, Ill.


### REPORTS OF CASES.

### EXCISION OF A MAMMARY GLAND.

By W. J. MARTIN, V.S.,
KANKAKEE, ILL.

THE patient, a black coach-mare, was found to be suffering from a carcinoma of the right mammary gland. As she had never had a colt the owner was unable to account for the appearance of the malady. The fistulous tracts had been incised some time previously by a surgeon, but no benefit had been derived from it. I advised excision, to which the owner readily consented. The mare being cast, chloroform was administered. The mare was then rolled upon her back, and held in this position by two assistants and sacks of straw. The glands were now thoroughly washed with a warm solution of bichloride of mercury, 1 to 1000. An elliptic incision was now made along each side of the diseased gland, and carried down to the base of the same. The whole of the diseased tumor was now removed *en masse* by first gently lifting the tumor upward and separating the adherent tissue from beneath with the finger and handle of the scalpel. After removal of the tumor the blood-vessels were ligated as met with. The cavity was now sponged out with the warm bichloride solution, then dusted with iodoform, and the edges brought together by interrupted sutures of chromicized catgut. The mare was now permitted to rise, when a roll of absorbent cotton was placed over the wound and held in place by a broad bandage around the body. The mare was now placed in a stall from which all straw and dust had been removed, and her head tied up to prevent her from lying down, and ordered to be fed with bran and hay for a few days. On the third day the bandage was removed, the wound dusted with iodoform as before, and the bandage reapplied. From this time the mare was exercised and permitted to lie down. She made a rapid recovery, and after a lapse of one year shows no sign of a return of the disease.

---

DR. L. R. WEBBER, of Rochester, N. Y., reports a case of intussusception in a gelding. When first noticed the animal was perspiring freely; abdomen distended with gas; would lie down, but did not try to roll. Looking upon the case as one of acute indigestion, it was treated as such with temporary relief for some ten hours, when all the symptoms of pain returned, with regurgitation of gas. All remedies at this time seemed powerless to give relief, though he lived forty-eight hours. Autopsy revealed the contents of stomach and small intestines fluid, the large intestines well filled with solid ingesta. The posterior three feet of the ileum was telescoped upon itself, and this part through the ileo-cæcal valve into the cæcum.

On to Buffalo!

